# Soluble sugar, organic acid and phenolic composition and flavor evaluation of plum fruits

**DOI:** 10.1016/j.fochx.2024.101790

**Published:** 2024-08-31

**Authors:** Qiao Xiao, Shiyuan Ye, Hao Wang, Shanshan Xing, Wenli Zhu, Haonan Zhang, Jiawei Zhu, Changbing Pu, Dongqi Zhao, Qiong Zhou, Jin Wang, Lijin Lin, Dong Liang, Xiulan Lv

**Affiliations:** a College of Horticulture, Sichuan Agricultural University, Chengdu 611130, China; b Hanyuan County Agricultural and Rural Bureau, Ya'an 625000, China

**Keywords:** Plum, HPLC, Fruit flavor, Sensory evaluation, Astringency, Rating criteria, Glucose (PubChem CID: 5793), Fructose (PubChem CID: 5984), Sorbitol (PubChem CID: 5780), Sucrose (PubChem CID: 5988), Malic acid (PubChem CID: 525), Quinic acid (PubChem CID: 6508), Citric acid (PubChem CID: 311), Succinic acid (PubChem CID: 1110), Epicatechin (PubChem CID: 72276), Gallic acid (PubChem CID: 370)

## Abstract

Plums (*Prunus salicina* and *Prunus domestica*) are prevalent in southwestern China, and have attracted interest owing to their delectable taste and exceptional nutritional properties. Therefore, this study aimed to investigate the nutritional and flavor properties of plum to improve its nutritional utilization. Specifically, we determined the soluble sugars, organic acids, and phenolic components in 86 accessions using high-performance liquid chromatography. Notably, glucose, fructose, malic, and quinic acids were the predominant sweetness and acidity in plums, with sucrose contributing more to the sweetness of the flesh than the peel. Moreover, The peel contains 5.5 fold more phenolics than flesh, epicatechin, gallic acid, and proanthocyanidins C1 and B2 were the primary sources of astringency. Correlation and principal component analyses showed eight core factors for plum flavor rating, and a specific rating criterion was established. Conclusively, these findings provide information on the integrated flavor evaluation criteria and for enhancing optimal breeding of plums.

## Highlights

• Glucose and fructose, malic and quinic acids, are the major sweet and sour flavoring substances in plums.
• Sucrose was the major contributor to the difference in sweetness of the flesh compared to the peel.
• Epcatechins, gallic acid, and proanthocyanidins C1, B2 are the main astringent factors.
• Plum is rich in phenolics and can be extracted as a functional food ingredient.

## 1 Introduction

Plum is a drupe fruit belonging to the subgenus *Prunus* of the family Rosaceae. Plum is one of the most widely distributed and cultivated fruit crop in the world, domesticated approximately 3000 years ago, and is mainly categorized into two types: hexaploid European plum (*Prunus domestica*) and diploid Chinese plum (*Prunus salicina*) (Deng et al., 2023). Recently, there has been increased appreciation for plum among consumers owing to its superior nutrition and flavor, attractive colors, and natural antioxidant and anti-inflammatory functional components (Lin et al., 2023). Additionally, plums are important in human diet and health because of their sugar, organic acid, phenolic, anthocyanin, carotenoid, mineral, and amino acid contents. Plums have been used in a variety of applications such as cooking, drying, and fruit wine making, and as dietary supplements and functional foods rich in bioactive compounds (Liaudanskas et al., 2020). In the past few years, the Chinese plum industry has developed rapidly and has become one of the most prominent industries in southwest China (Liu, 2018). Notably, plums show a rich and wide diversity of traits, such as fruit size, color, shape, and texture, flavor, and physiological characteristics (Milošević & Milošević, 2018; Milošević, Milošević, & Mladenović, 2023).

Flavor is one of the most essential characteristics that directly affects market competitiveness, and is affected by the sugar, organic acid, amino acid, and secondary metabolite composition. Importantly, the level and proportion of flavor-related traits (soluble sugars and organic acids) are key indicators of the quality and flavor of plums. For example, the superior sugar-sour taste strongly depends on the abundance of specific primary metabolites (sucrose, glucose, sorbitol, fructose, citric acid, malic acid, and tartaric acid), as well as the total sugar and acid contents. Generally, fruit ripening tends to decrease the organic acid content and increase the sugar content (Zhou et al., 2018). Based on the accumulation characteristics at maturity, soluble sugars are classified as fructose, glucose, sucrose, sorbitol, and other accumulation types. Considering that each carbohydrate has a different sweetness, sweetness values can be calculated based on relative quantity and sweetness properties: if glucose is rated 1, then fructose is 2.3, sucrose is 1.35, and sorbitol is 0.81 times sweeter than glucose (Roussos, Sefferou, Denaxa, Tsantili, & Stathis, 2011). Notably, malic acid is the major organic acid in plum (Yu et al., 2023) and pear (Wu et al., 2022), whereas tartaric and quinic acids are the main organic acids in grape (Zhao et al., 2023) and kiwifruit (Wang et al., 2022), respectively. Importantly, these acids contribute to acidity and sweet flavor of fruits. Sugars and organic acids have been the topic of numerous studies on fruit flavor (Lin et al., 2023; Liu et al., 2024). Currently, investigations on plums are mainly focused on the mechanism of sugar and organic acid accumulation and metabolism during fruit development (Lo Piccolo et al., 2021; Nie, Hong, Wang, Lu, & An, 2023; Yu, Ali, Li, Fang, & Chen, 2021), with limited studies on the classification and evaluation of sweet-sour flavors.

Among members of the Rosaceae family, plums have the highest total phenol content (TPC), total antioxidant capacity (TAC), total flavonoids content (TFC), 2,2′-diphenyl-1-picrylhydrazyl (DPPH), ferric reducing antioxidant power (FRAP), and 2,2′-azinobis-(3-ethylbenzo-thiazoline-6-sulfonic acid (ABTS) values, followed by peaches and pears (Hameed et al., 2022).Research findings indicate that the TPC and TAC of plum peel tissue are 6.6-fold higher than that of the flesh, and their contents vary considerably among cultivars (Drogoudi & Pantelidis, 2022). Phenolic compounds are critical secondary metabolites in plants with potent anti-oxidant activities that significantly reduce the risk of diabetes, neurological disorders, and certain cancers (Marchiosi et al., 2020). Moreover, these compounds tend to affect pigmentation and impart bitterness, astringency, and a phenolic-like taste, thus enhancing the flavor (Zhang et al., 2024).

Variations in carbohydrates, organic acids, and phenolics have been shown to cause differences in fruit flavor (Zou et al., 2020).Flavor compounds differ not only among different species, but also among different organs and tissues within a species (Drewnowski & Gomez-Carneros, 2000). However, the relationship between sugars, acids, and phenolics in different tissues and the flavor of plums remain unclear. Different from previous studies, we did not limit to using just the evaluation indexes such as total soluble solids, solid-acid ratio and titratable acid to assess the flavor profile of plums, but also took into full consideration the contribution of specific flavor substance components, and not only that, we also compared the flavor substances in different tissues in order to have a more comprehensive understanding of the true flavor of plums. Although the method we used did not cover all flavor substance types, a comprehensive analysis was also done to evaluate plum flavor in terms of soluble sugars, organic acids and phenolic compounds as well.

In order to investigate how the type and content of sugars, acids and phenolics jointly affect the flavor profile of plums, we examined sugars, organic acids and phenolics in peel and flesh of 86 plum accessions comprising different genotypes, sizes, shapes and colors, as well as fruit ripening stages. Overall, the objectives of this study were to characterize the soluble sugar, organic acid, and phenolic components and contents of various plum accessions; evaluate the effects of major sweet, sour, and astringent compounds on plum flavor; and establish rating criteria for plum flavor evaluation. Results of the study may provide a reference for plum fruit flavor evaluation, nutrition utilization and breeding improvement.

## 2 Materials and methods

### 2.1 Plant materials

In total, 86 plum accessions were collected in southwest China including Sichuan, Guizhou, Yunnan and Chongqing provinces, and additional information is shown in Table S1. Approximately 40 fruits were randomly harvested from each batch of samples, and the physiologically mature stage was comprehensively judged based on indicators such as soluble solids, fruit color, and fruit size. All samples were randomly and equally divided into three parts for sensory evaluation, ripening index analysis, and determination of related substances using HPLC. For HPLC, the peel and flesh of the fresh fruits (10 fruits per replicate) were separated, immediately frozen in liquid nitrogen, and stored at ˗80 °C.

### 2.2 Sensory evaluation

A taste panel of 10 experts assessed the flavors according to Karagiannis (Karagiannis et al., 2021) with modifications. Assessors confirmed the sweet-sour flavor (sour, sour-sweet, sweet-sour, sweet) and scored each accession according to sweet-sour ratings (sour, 1.0–2.5; sour-sweet, 2.6–4.0; sweet-sour 4.1–5.5; sweet, 5.6–7.0). Additionally, the astringency of the fruits was rated as none (0), light (1.0), medium (2.0), and heavy (3.0), based on the consensus of all assessors. Flavor ratings were calculated by the final score = (total score ˗ the highest score ˗ the lowest score)/8, and the average score is shown in Table S1. All members were fully informed of the purpose, process, and allergen information prior to the evaluation. Standardized characterization and evaluation of plum color and fruit shape were performed according to the UPOV (International Union for the Protection of New Varieties of Plants) (Drogoudi & Pantelidis, 2022). Ethical permission was not required for this study, and all participants agreed to take part in the sensory study and to use their information.

### 2.3 Ripening index analysis

Briefly, fruit size (g FW) was measured using an electron balance with 0.01 g precision (JM-A3003, TP-001, China). Total soluble solid (TSS, ^°^Brix %) was measure in freshly prepared fruit juice using a digital hand refractometer (ARP-TD32; Airui Pu, China). Notably, the final fruit size and TSS were based on the averages of 10 fruits per sample. Titratable acidity (TA, malic acid %) was measured by titrating the fruit juice against 0.02 mol/L NaOH solution. Index measurements were performed using three biological replicates.

### 2.4 Extraction and detection of sugar, organic acid, and phenolic contents

The extraction, determination, and quantification of soluble sugars (Yao et al., 2022), organic acids (Lin et al., 2023) and phenolic compound (Zhang et al., 2024) were performed based on a previously described method as slightly altered. Briefly, fresh samples were ground into powder in liquid nitrogen. Thereafter, approximately 1.0 g of the powder was extracted twice with 5 mL of ultrapure water at 80 °C for 15 min at each time to obtain the soluble sugars. For the organic acid determination, 1.0 g of powder were extracted twice with 5 mL of 0.2 % phosphoric acid solution (pH = 2.6) for 20 min at each time, followed by centrifugation at 10,000 r/min for 10 min at 4 °C. Finally, the supernatants from each sample were combined for further analysis. For phenolic compound extraction, 0.5 g of powder were extracted with 1.5 mL of a solution [V (methanol): V (water): V (formic acid) =70:28:2] for 30 min, followed by centrifugation at 8000 r/min for 15 min at 4 °C. Thereafter, 1 mL of the supernatant was filtered with a 22-μm microporous nylon syringe for HPLC.

Soluble Sugars were detected using an Agilent G1362A refractive index detector (RID) with a reference cell maintained at 40 °C. Sugar separation was performed using a Thermo NH_2_ column (4.6 mm × 250 mm, 5 μm) at 40 °C. The mobile phase consisted of acetonitrile: water (80,20, *v*/v) at a flow rate of 1.0 mL/min, with injection volume of 10 μL. Organic acids were analyzed within an Agilent G1314F Variable Wave­length detector (VWD) equipped with a Comatex C18 column (4.6 mm × 250 mm, 5 μm) maintained at 25 °C. The mobile phase consisted of 3 % methanol and 97 % phosphoric acid solution, with an injection volume was 20 μL and flow rate of 0.8 mL/min. The UV absorbance was detected at 210 nm. Phenolics were detected using an Agilent G1314F VWD coupled with a C18 column maintained at 30 °C. The mobile phase consisted of 2 % formic acid solution and acetonitrile, with injection volume of 20 μL and flow rate of 1 mL/min. The UV absorbance was measured at 367, 320, and 280 nm. The results were expressed in mg/g FW for sugars and mg/100 g FW for organic acids and phenolics. The contents of all analyzed sugars, acids, and phenolics were summarized and submitted as total sugars, acids, and phenolics.

### 2.5 Calculation of sweet index, sugar-acid ratio, and sweetness-acid ratio

The sweetness index (SI) was calculated based on the relative content and sweetness attributes of different sugar fractions as previously described (Roussos et al., 2011). SI = fructose content × 2.3 + sucrose content × 1.35 + glucose content × 1 + sorbitol content × 0.81. The sugar-acid ratio was defined as the proportion of total sugars and total organic acids in the sample, and the sweetness-acid ratio was the ratio of SI to total acid.

### 2.6 Statistical analysis

All statistical analyses were performed using Microsoft Excel 2016 and SPSS 27.0 (IBM, Armonk, NY, USA). Statistical comparisons were performed using ANOVA, followed by Duncan's multiple range test. Statistical significance was set at *p* < 0.05. Bar and box charts were generated using the GraphPad Prism 8.0 (GraphPad Software, San Diego, CA, USA). Correlation and principal component analysis were using Spearman's rank method The Kolmogorov–Smirnov test was used to test the normality of the indices. Hierarchical clustering was performed using Ward's linkage and squared Euclidean distance method. All data are presented as mean values ± standard deviation.

## 3 Results

### 3.1 Composition of soluble sugars in plum accessions

**Fig. 1:**
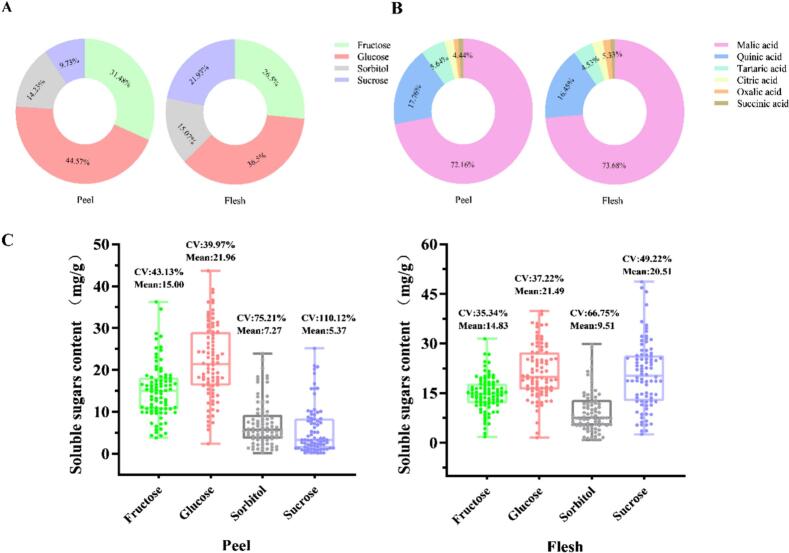
Proportion of sugars in the peel and flesh of 86 plum accessions. (A, B) Proportion of soluble sugars and organic acid. (C) The contents of different sugars in the flesh and peel of plums. The horizontal lines in the interior of the box are mean values. Each scatter plot indicates the amount of each substance. Cv, coefficient of variation.

Notably, four soluble sugars were identified in the plum accessions using HPLC (Fig. 1A and C). Among the sugars, glucose accounted for 32.39 and 44.57 % of the total sugar in the flesh and peel, respectively, with the contents ranging from 1.50 to 39.76 and 2.36–43.63 mg/g FW, respectively. Additionally, fructose accounted for 31.48 % of total sugar, with contents ranging from 3.78 to 36.22 mg/g FW. Importantly, there were no significant differences in the concentrations of fructose and glucose between the flesh and peel. In contrast, sucrose was significantly higher in the flesh (average of 3.82-fold higher) than in the peel (Fig. S1). Sucrose was second only to glucose in the flesh, with a maximum content of 48.64 mg/g (SYC) and an average content of 20.51 mg/g FW. Sorbitol was the least abundant, accounting for only 15.07 % (0.86–29.81 mg/g FW) and 14.23 % (0.43–23.89 mg/g FW) of total sugar in the flesh and peel, respectively. Among the 86 accessions, the coefficients of variation (Cv) for glucose and fructose were comparatively small compared to that of other sugar fractions, 35.34 and 43.13 % for glucose and 37.22 % and 39.79 % for fructose in the flesh and peel, respectively. It indicates that the two main sugar components in plum fruits are well stabilized. Overall, glucose and fructose were the main contributors to the sweet flavor. However, the Cv for sorbitol and sucrose were high, 66.75 and 75.21 % for sorbitol and 49.22 and 110.12 % for sucrose in the flesh and peel, respectively. Sucrose had the highest Cv in the peel and showed the greatest diversity among accessions (Fig. 1C). Notably, the average total sugar contents of the flesh and peel were 60.93 and 49.59 mg/g FW, respectively, with average SI of 90.99 and 69.58, respectively (Table S2). And sucrose might be the main reason for the difference in sweetness between peel and flesh. Overall, the sugar content of the flesh was stable compared to the peel and contributed considerably to the sweet flavor of the fruit, which was confirmed by the sweetness index.

### 3.2 Composition of organic acids in plum accessions

**Fig. 2:**
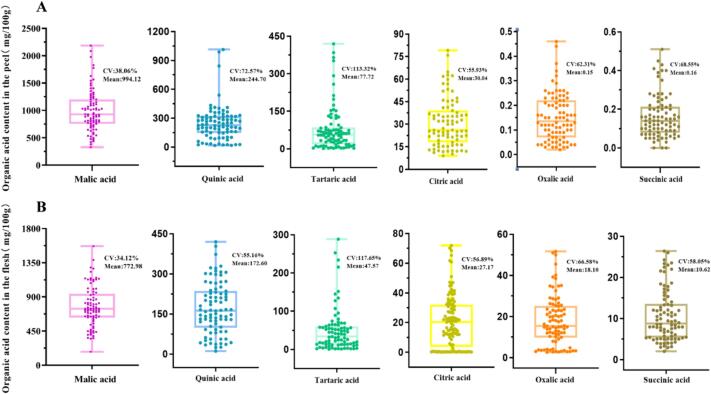
Distribution of organic acids in the peel and flesh of 86 plum accessions. The horizontal lines in the interior of the box are mean values. Each scatter plot indicates the amount of each substance. Cv, coefficient of variation.

In the present study, six organic acids were identified in all accessions (Fig. 1 B). Notably, malic acid was the most abundant compound, accounting for an average of 73.70 % (177.58–1568.43 mg/100 g FW) and 72.17 % (327.68–2187.46 mg/100 g FW) of the total acids in the flesh and peel, respectively. Additionally, quinic acid accounted for an average of 16.44 % (11.00–420.31 mg/100 g FW) and 17.75 % (16.33–1012.48 mg/100 g FW) of total acids in the flesh and peel, respectively. Moreover, we detected slight amounts of tartaric (0.70–288.40 and 1.09–419.47 mg/100 g FW), citric (2.70–71.96 and 9.03–79.14 mg/100 g FW), oxalic (2.66–51.69 and 2.15–45.63 mg/100 g FW), and succinic acids (2.04–26.41 and 2.97–51.37 mg/100 g FW) in the flesh and peel, respectively. Overall, the ratio of malic acid to quinic acid and the remaining trace acids in the plums was 7:2:1. Furthermore, there were significant differences in the concentrations of tartaric, quinic, and malic acids between the peel and flesh. In contrast, there was no significant difference in the concentrations of the other compounds, accounting for only 5.33 and 4.44 % of the total acid in the flesh and peel, respectively. Importantly, the organic acids had a high Cv ranging from 34.19 to 117.62 % and 38.00–113.32 % in the flesh and peel, respectively. Malic acid, which was the dominant organic acid, had the smallest Cv of 34.19 and 38.0 % in the flesh and peel, respectively (Fig. 2A and B). It indicates that the predominant organic acids in plums are relatively stable. Overall, these data indicate that organic acids may contribute more to flavor quality of plum than soluble sugars. Notably, the average total acid contents of the flesh and peel were 1049.81 and 1378.39 mg/100 g FW, respectively. Moreover, the flesh had a higher sweetness to acid and sugar to acid ratio than the peel (Table S3), indicating that the higher acidity of the peel contributed to a more pronounced acidic flavor. Combined with the distribution of soluble sugars, more acid and less sugar accumulated in the peel resulting in a more acidic flavor.

### 3.3 Composition of phenolic in plum accessions

**Fig. 3:**
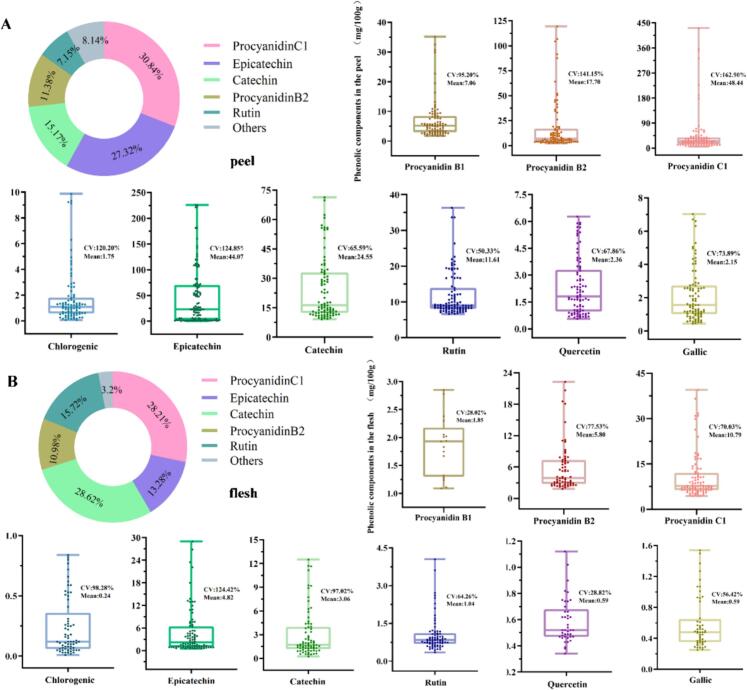
Proportion and distribution of phenolic compounds in the peel and flesh of 86 plum accessions. Proportion and distribution of phenolic compounds in the peel (A) and flesh (B). Others in circle indicate procyanidin B1, chlorogenic, quercetin, gallic. The horizontal lines in the interior of the box are mean values. Each scatter plot indicates the amount of each substance. Cv, coefficient of variation.

Nine monomeric phenols were detected in the accessions and divided into three main groups: flavanols (proanthocyanidins B1, B2, C1, catechin, and epicatechin), flavonols (rutin and quercetin), and phenolic acids (gallic acid and chlorogenic acid) (Fig. 3A and B). Flavanols were the most abundant phenols, accounting for 91.46 and 88.81 % of the total phenol contents of the flesh and peel, respectively. Among flavanols, proanthocyanidins C1(PAsC1) was the most abundant with an average of 10.79 and 48.44 mg/100 g FW in the flesh and peel, respectively, followed by epicatechin with an average of 4.82 % and 44.07 % mg/100 g FW, catechin with an average of 3.06 and 24.55 mg/100 g FW, proanthocyanidins B2 (PAsB2) with an average of 5.80 and 17.70 mg/100 g FW, and proanthocyanidins B1(PAsB1) with an average of 1.85 and 7.06 mg/100 g FW, respectively. Phenolic acids and flavonols only accounted for 8.54 and 11.19 % of phenolic compounds in the flesh and peel, respectively, with gallic acid accounting for 0.59 and 2.15 mg/100 g FW, chlorogenic acid accounting for 0.24 and 1.75 mg/100 g FW, rutin accounting for 1.04 and 11.61 mg/100 g FW, and quercetin accounting for 0.59 and 2.36 mg/100 g FW, respectively.

Among the 86 materials, all nine monomeric phenols were detected in the peel, whereas only PAsC1 and epicatechin were detected in the flesh (Table S4). Almost all phenolic compounds were more abundant in the peel than in the flesh. Collectively, these data indicated that the major phenolic compounds in the flesh and peels of plums were PAsC1, followed by epicatechin (0.24–12.51 and 9.16–71.34 mg/100 g FW) and catechin (0.49–28.98 and 0.54–225.90 mg/100 g FW). In addition to the three major phenolic components, the PAsB2 contents of the flesh and peel ranged from 1.84 to 22.29 and 2.29–119.48 mg/100 g FW, respectively, whereas that of rutin ranged from 0.34 to 4.05 and 6.64–36.33 mg/100 g, respectively. Importantly, the remaining detected phenolics were present only in trace amounts, with mean concentrations below 10 mg/100 g FW. Moreover, the levels all detected phenolic monomers in the tissues and accessions showed instability, with values ranging from 50.33 to 162.90 % and 28.02–124.42 % in the peel and flesh, respectively. Furthermore, the average total phenolic contents of the flesh and peel were 28.79 and 159.71 mg/100 g FW, respectively, with the total phenolic content of the peel 5.55-fold higher than that of the flesh. Notably, the highest level of the peel (ALS, 728.52 mg/100 g of peel) reached nearly 10-fold that of the flesh (75.83 mg/100 g).

### 3.4 Comprehensive assessment of the flavors of plum accessions

**Fig. 4:**
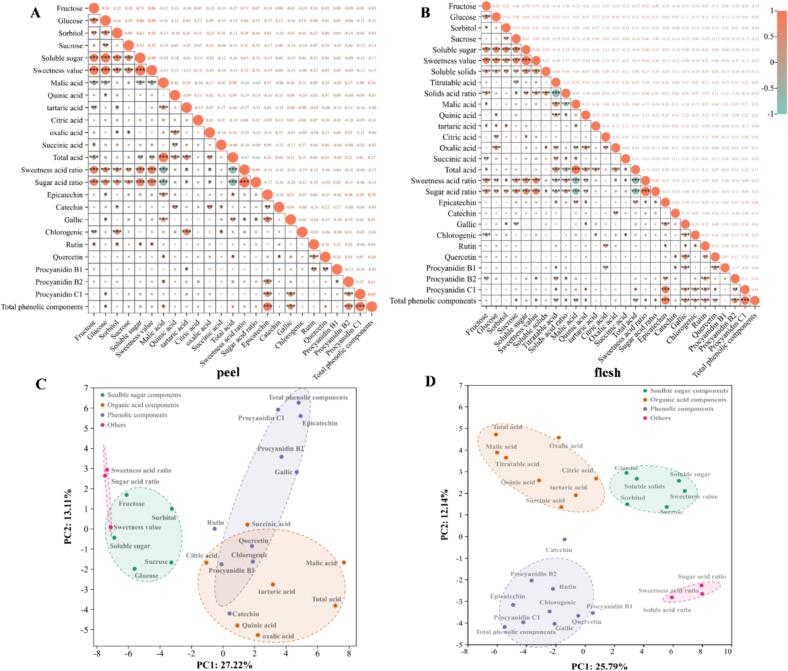
Correlation analysis (A, B) and principal component analysis (PCA) (C, D) of all assessed variables for sugars, organic acids, and phenolic compounds in 86 plum accessions. A, C represents peel and B, D represents flesh. *, **, and *** indicate significance at 0.05, 0.01, and 0.001 level, respectively.

To further investigate the relationship between the plum flavor variables, a correlation analysis was performed (Fig. 4A and B). Notably, the trend of correlations between the individual flavor components in the peel were highly comparable to that in the flesh, suggesting that the flesh and peel play similar roles in contributing to plum flavor. Soluble solids were significantly positively correlated with fructose (0.38^⁎⁎⁎^), glucose (0.36^⁎⁎⁎^), sorbitol (0.34^⁎⁎^), sucrose (0.29^⁎^), soluble sugar (0.53^⁎⁎⁎^), and SI (0.53^⁎⁎⁎^). In contrast, solid-acid ratio was significantly negatively correlated with titratable acid content (˗0.83^⁎⁎⁎^). Among the identified organic acids, malic acid had the highest correlation with total acids (0.94^⁎⁎⁎^) and a significant negative correlation with solid-acid ratio (˗0.42^⁎⁎⁎^). Additionally, the total phenolic compounds in the peel and flesh showed a high positive correlation with epicatechin, gallic acid, PAsB2, and PAsC1. Moreover, a positive correlation was observed between total acids and titratable acid (0.28**), malic acid (0.31**), and phenols (0.31**). Overall, these results indicate that acidity is related to astringent flavors in plums.

Furthermore, we assessed the variation in flavor indicators in plums using principal component analysis (Fig. 4C, D). Notably, the two principal components (PC1 and PC2) contributed 37.93 % (25.79 and 12.14 %, flesh and peel, respectively) and 40.33 % (27.22 and 13.11 %) to the total variation in the flavor compositions of the flesh and peel, respectively. Additionally, the multiple indicators formed four distinct clusters. Notably, sugars, acids, and phenolics were clearly separated, whereas the internal components of each group clustered together. Based on the previous results, the sugar-acid flavor of plum can be assessed using the following indices: soluble solids, fructose, glucose, sucrose, titratable acid, malic acid, and solid acid ratio. Additionally, the indices for assessing astringency were simplified to total phenolics.

### 3.5 Rating system construction and cluster analysis

Total soluble solids, titratable acid, solid-acid ratio all varied widely among the 86 plum accessions, total soluble solids ranging from 7.77 % (QCA) to 20.27 % (WD). TA ranging from 0.32 %(ALS)–1.65 %(QMG), and solid-acid ratio ranging from 5.61 %(HJG)–35.07 %(QF), respectively. Based on the solid-acid ratio ranges, we classified the plum flavor into four clusters (Table S1): sour, sour-sweet, sweet-sour, and sweet. Additionally, we compared this categorization with taste ratings and found that all examined materials conformed to the grade classification (Table S8); however, there were significant differences between the two evaluations (*p* < 0.03*). Owing to these differences, we digitally quantified eight key traits and found that soluble solids, fructose, glucose, sucrose, TA, and the TSS/TA ratio followed a normal distribution. However, malic acid and total phenolic content did not follow this trend (Fig. S2). Sweet-sour flavor was classified into five grades (Table 1) by querying the data of traits corresponding to z-values of 0.1, 0.3, 0.7, 0.9 in the z-value respectively, and astringency was divided into four evaluation levels (Table 2). Accessions with astringency were predominantly light (*p* < 2). Correlation analysis showed that total phenolic content was significantly correlated with astringency in the peel (0.71**) and flesh (0.42**). Notably, epicatechin had the highest correlation (0.64**) with astringency, followed by gallic acid (0.48**), proanthocyanidin C1 (0.42**), and proanthocyanidin B2 (0.36**, Table S9).

**Table 1:** Detailed rating in sweet-sour flavor of plums.

Index	Rating (Solid-acid ratio)				
	1 (<9.0)	2 (9.0–13.0)	3 (13.0–20.0)	4 (20.0–26.0)	5 (≥30.0)
TSS (%)	<9.4	9.4–11.0	11.0–13.0	13.0–15.0	≥15.0
TA (%)	<0.5	0.5–0.7	0.7–1.0	1.0–1.2	≥1.2
Fructose (mg/g)	<9.0	90.–12.0	12.0–18.0	18.0–22.0	≥22.0
Glicose (mg/g)	<13.0	13.0–18.0	18.0–26.0	26.0–32.0	≥30.0
Sucrose (mg/g)	<13.0	13.0–17.0	17.0–27.0	27.0–34.0	≥34.0
Malic acis (mg/100 g)	<480.0	480.0–650.0	650.0–910.0	910.0–1110.0	≥1110.0

**Table 2:** Detailed rating in astringency of plums.

Type	Rating			
Total phenolic contents (mg/100 g)	15.0–100	100–230	230–320	≥320
Astringency values	1	2	3	4

**Fig. 5:**
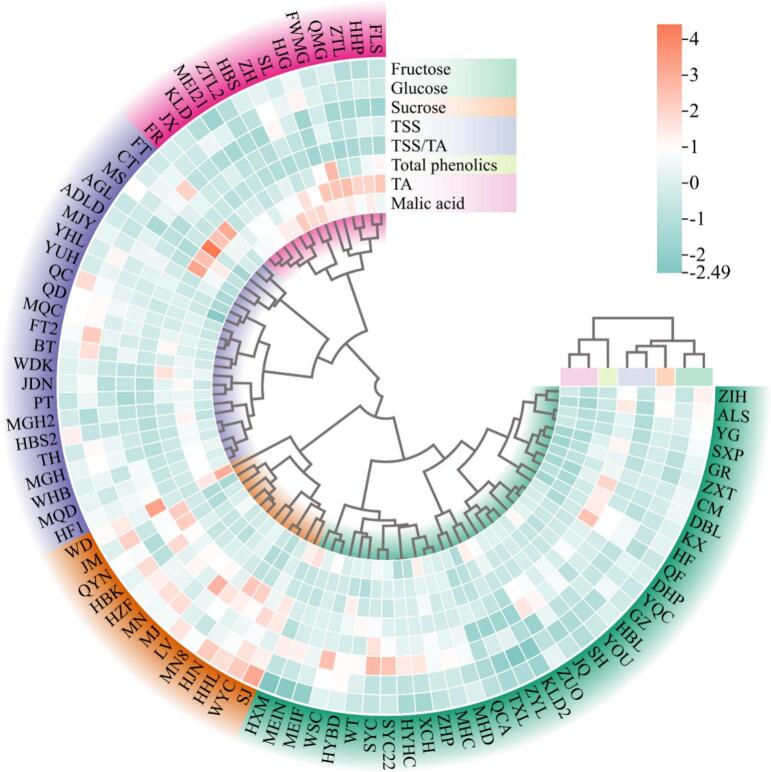
Hierarchical cluster analysis of plum accessions based on eight flavor indexes.

Furthermore, the 86 accessions were classified into four clusters following hierarchical cluster analysis based on the eight flavor indicators (Fig. 5). Importantly, Cluster I comprised 36 accessions (41.86 % of all accessions), Cluster II comprised 13 varieties (15.12 %), Cluster III comprised 23 accessions (26.74 %), and Cluster IV contained 14 accessions (16.28 %). A calculation of the average of the eight flavor evaluation criteria for each cluster (Table S10) showed that the salient features of the resources in Cluster I were high sucrose content, low titratable acid content, and high solid-acid ratio. Therefore, this cluster was defined as a sucrose-dominant type. Additionally, majority of plums belonged to Cluster I, which was characterized by high sucrose content during fruit ripening. Cluster II was characterized by a higher glucose content compared with fructose and sucrose content, a higher concentration of all types of sugars, solids, and solid acid ratios, and moderate titratable acid, defining this cluster as a comprehensive high-sugar type. The varieties in this group generally have relatively high flavor values. Cluster III was labelled as having the highest phenolic content and moderate soluble solids, titratable acid, and solid acid ratios, which defined this cluster as a phenolic-dominant type. Cluster IV was characterized by high acid and low sugar and phenolic contents in the middle to upper range. Consequently, this group was defined as the hyperacidic type, and included European plums, which are characterized by large fruit size, dark red or purple-black color, and sour-astringent taste.

## 4 Discussion

### 4.1 Soluble sugars, organic acids, and phenolics in plums

Fruit flavor is mainly affected by the type and proportion of sugar-acid components. Notably, the compositions of soluble sugars and organic acids vary widely among fruit crops and appear to diversify during plum growth. Our study showed that plums are mainly classified as fructose and glucose type, sucrose type and a small amount of sorbitol accumulating type, different types of accumulated sugar substances give plums a diverse sweet flavor profile, which confirmed the previous conclusions. (Nie et al., 2023; Tomić, Štampar, Glišić, & Jakopič, 2019; Yao et al., 2022). Sorbitol, which is commonly used as a sugar substitute and has mild laxative and protective functions, was detected in all examined plum accessions. Diets consisting of glucose and sorbitol generate a minor glycemic burden, probably owing to the inhibition of glucose production in the presence of sorbitol (Stacewicz-Sapuntzakis, 2013). Therefore, the health significance of plums for individuals with diabetes and cardiovascular diseases cannot be overemphasized. According to the type of soluble sugar content, the predominant soluble sugar in the plums was glucose, and was assumed to be present in the largest proportion in the different tissues. It was closely followed by fructose. Additionally, the flesh had similar sucrose and glucose contents, but with no significant difference in levels. Moreover, the peel mainly contained glucose and fructose, with relatively low concentrations of sucrose. Overall, these results suggest that fructose and glucose are responsible for the sweet flavor in plums and that sucrose is the main cause of the difference in sweetness index between the flesh and peel.

The sugar-acid balance is a key determinant of fruit flavor, and acidity contributes to a better taste. Consistent with findings in five plum cultivars in Sichuan (Lin et al., 2023), malic acid and quinic acid were the most dominant acids in the plum accessions. Moreover, a previous study showed that quinic acid was the principal marker in local plum cultivars in Lithuania (Liaudanskas et al., 2020). Quinic acid is a precursor for aromatic amino acid biosynthesis and contributes to the flavor of plant-derived foods by regulating the synthesis of aromatic compounds (Ghosh, Chisti, & Banerjee, 2012). In the present study, the ratio of malic acid to quinic acid and other trace acids was approximately 7:2:1, which may contribute to the characteristic acidity of plum. Additionally, we showed that that organic acids in plums had a greater influence on the sweet-sour flavor than soluble sugars. Similarly, acidity rather than sweetness may have been selected during apple (Ma et al., 2015) and Chinese cherry domestication(Zhou et al., 2023), and selection for fruit acidity has a significant influence on fruit taste (Liao et al., 2021). Organic acids are not only essential for flavor improvement but are also more stable during storage and processing than other traits such as color and volatile compounds. As a renewable source of alternative chemical products, organic acids have been widely used in the food, pharmaceutical, and bio-based materials industries (Liu, Li, Liu, Du, & Chen, 2017). Notably, the distribution of organic acids in the peel and flesh was almost the same. Additionally, the higher levels of organic acids in the peel than in the flesh may be due to the translocation of organic acids with a defense function to the flesh, as well as the dilution effect of the expanded flesh, which leads to a richer taste sensation in the peel.

Consistent with findings in selected Serbian plum cultivars, flavanols were the most abundant phenolic compounds in the plum accessions. Contrary to findings in Serbian cultivars that showed that proanthocyanidin B1 was the most abundant flavanol, followed by proanthocyanidin B2, epicatechin, and procyanidin trimer (Tomić et al., 2019), proanthocyanidin C1 was the most abundant flavanol in this study, followed by epicatechin and catechin. This discrepancy may be because the materials used in the previous study were only European plums, whereas we combined different genotypic resources, such as *Prunus domestica*, *Prunus salicina*, *Prunus cerasifera*, and *Prunus simonii*m*.* Additionally, it may also be because the amount of proanthocyanidin C1 was strongly influenced by cultivar factor (η2 = 0.989, *p* < 0.001) (Liaudanskas et al., 2020). Proanthocyanidins, also known as condensed tannins, are a source of plant color and astringency, and are strong bioactive food-borne compounds with promising applications in the fields of pharmaceuticals, food, and cosmetics. Research in aronia berry juice has revealed that proanthocyanidins are the core astringent compounds (Huang, Fang, Xie, Liu, & Xu, 2022). Proanthocyanidins are polymers formed by the structural condensation of flavan-3-ol units, such as catechin or epicatechin, in varying amounts. UPLC-Q-Exactive Orbitrap/MS analysis detected 17 phenolic compounds in plums (Yu et al., 2021), with most of the free phenols being epicatechin, neochlorogenic acid, and proanthocyanidin B2, and the bound phenols being mainly catechin and epicatechin. In the present study, we found that epicatechin and catechin were abundant in plums. Importantly, the highest epicatechin content was detected in peel of ZTL (222.94 ± 0.77 mg/100 g FW) and the highest catechin content was found in JDN (71.34 ± 0.44 mg/100 g FW), with both compounds being present in trace amounts in the flesh. Catechins and epicatechins have strong astringent flavors and are commonly found in tea, cacao, wine, pears, and apples. Notably, oxidized phenols bind to proteins and reduce allergenicity. Catechins and epicatechins, which exist naturally in apples as substrates for polyphenol oxides, have been found to reduce allergenic Mald1 levels (Kiewning, Wollseifen, & Schmitz-Eiberger, 2013). Generally, the flavanol content of plums varies considerably between species and tissues, and plays a prominent role in human health and fruit flavor.

Consistent with the low levels of chlorogenic acid in 18 Serbian cultivars (Tomić et al., 2019), only trace amounts of gallic and chlorogenic acids, rutin, and quercetin were detected in the accessions examined in this study. Hameed et al. (2022) found that the plum cultivar Queen Rosa had a higher chlorogenic acid content (11.86 mg/100 g) than pears and peach (Hameed et al., 2022). In thecurrent research, the highest chlorogenic acid content in the peel of HJG was 11.23 mg/100 g FW. In contrast, a previous study showed that chlorogenic acid was the most plentiful phenolic compound in 17 plums (Liaudanskas et al., 2020). Norwegian researchers detected only slight amounts of flavonols (rutin and quercetin 3-glucoside) in six varieties of plum. According to Serbian scholars, the major flavonoid in plums was rutin (quercetin-3-rutinoside), with the concentrations of the other identified flavonols <0.50 mg/100 g FW (Tomić et al., 2019). In the present study, the average values of the three phenolics were low (< 3.0 mg/100 g FW), except for rutin. Although we characterized only two flavonoids, rutin was the dominant flavonoid. Importantly, the low levels of quercetin in the plums may be because most flavonoids are present in the plant as glycosides (Jäger & Saaby, 2011). Therefore, further in-depth analyses of the effect of glycosides on the flavor of plum fruit are needed.

### 4.2 Flavor evaluation of plum accessions

In horticulture, soluble solids, titratable acid content, and solid acid ratio are typically employed to assess fruit flavor and ripening. Generally, excessively high titratable acid content can restrict consumer preference. Consistent with values in five Sichuan plums (0.82–1.38 %) (Lin et al., 2023) and 43 European and Japanese plums (0.5–1.9 %) (Drogoudi & Pantelidis, 2022), titratable acid ranged from 0.32 to 1.65 % in the 86 accessions in the present. Moreover, soluble solids were above the minimum value for certain drupe fruits in the European Union (8° Brix), and the higher solid-acid ratios represents a more intense fruit flavor (Table S1). However, the solid and solid-acid ratios are not perfect predictors of plum flavor. Sweet fruits are not necessarily high in sugar levels, but may be low in organic acids, particularly malic acid, such as ALS and GR. Additionally, each compound has a different taste threshold and type, making it more appropriate to combine individual flavor components to evaluate plum flavor. Considering that sugars and acids are basic flavor compounds, it is necessary to identify other flavor chemicals, such as amino acids, volatiles, and alkaloid (Cao et al., 2022), using cutting-edge techniques such as UPLC-ESI-MS/MS.

In many food matrices, astringency is closely related to phenolic compounds (Huang & Xu, 2021), In the present study, the prominent astringency was attributed to the significant correlation between astringency value and total phenolic compounds in the peel. Epicatechin, gallic acid, and proanthocyanidins C1 and B2 may be the major astringent compounds present in fresh plums. Some astringent substances in plums are condensed tannins, which are relatively weak in astringency compared with hydrolyzed tannins, resulting in a smooth and refreshing mouth sensation, light roughness, and a wrinkled feeling. Consumers reacts differently to astringent flavors, and taste factors, such as genetics, sex, and age may affect consumers' acceptance of astringent plant foods. Therefore, methods, such as the salivary precipitation index and mucin turbidity (Huang et al., 2022) should be applied to evaluate astringency in fresh plums, and more stringent identification and verification should be carried out using thiolysis and LC-MS/MS.

Conclusively, eight flavor traits were identified for plum flavor evaluation, namely soluble solids, fructose, glucose, and sucrose as sweetness factors; malic acid and titratable acid as acid factors; solid-acid ratio as sugar-acid overall flavor; and total phenolics as astringent flavor. Specific grading criteria for the flavor quality of plums were formulated and provide theoretical references for the efficient utilization of plum germplasm resources and genetic breeding.

### 4.3 Characteristics of different accessions and application recommendations

In the present study, we examined 86 plum accessions comprising different genotypes (*P.salicina*, *P.domestica, P.simonii*, *P.cerasifera atropurpurea*), size range (13.5–135.0 g), shape and color (yellow, green, red, dark purple), and fruit ripening stage (late May – early September). Based on hierarchical cluster analysis of eight indicators, the 86 plum accessions were classified into four groups: sucrose-dominant, comprehensively high-sugar, comprehensively high-phenolic, and hyperacidic, which systematically recognized the diversity of flavors among the different accessions. Most plums were characterized as the sucrose-dominant type, followed by the comprehensively high-phenolic type. Importantly, 13 accessions of the comprehensive high sugar type, such as WD/WYC/LV/MN, and 23 accessions of the comprehensive high phenolic type, such as QC/MQC/QD/FT/HF, may be suitable for popularization because of their high quality and nutritional value. Parental selection can also be made according to the target traits during the selection and breeding of new varieties. For example, QMG, which contains abundant phenolics and is a unique resource with a dark red peel and flesh color, can be used as a high-acid parental material in crossbreeding. According to the flavor characteristics, different plum resources are rationally applied to diverse industries.

## 5 Conclusion

In this study, sugar, acid and phenolics of plum fruits were combined with sensory evaluation to assess the flavor profile comprehensively for the first time. We showed that the main soluble sugars in plum accessions from Southwest China were glucose and fructose, with the proportion varies by varieties, and that the difference in sweetness between the peel and the flesh depended on the sucrose content. Additionally, malic and quinic acids were the main organic acids in the accessions, and organic acids in plums had a greater influence on the sweet-sour flavor than soluble sugars. Phenolic compounds mainly concentrated in the peel, which was 5.5 fold higher than that in flesh. Moreover, the main substances affecting astringency were epcatechins, gallic acid, and proanthocyanidins C1, B2. Eight core indices for plum flavor rating were obtained through principal component and correlation analyses, including soluble sugar, glucose, sucrose, fructose, titratable acid, malic acid, solid-acid ratio, and total phenolics. Plum flavor grading criteria were formulated according to eight evaluation indicators to provide a guiding method for assessing plum fruit flavor. The results of this study will lay the foundation for flavor evaluation and quality improvement of plum germplasm resources.

## Funding information

This work was supported by Ya'an Science and Technology Program (22CGZHZF0005).

## CRediT authorship contribution statement

**Qiao Xiao:** Writing – review & editing, Writing – original draft, Data curation, Conceptualization. **Shiyuan Ye:** Validation, Software. **Hao Wang:** Investigation, Data curation. **Shanshan Xing:** Data curation. **Wenli Zhu:** Formal analysis. **Haonan Zhang:** Methodology. **Jiawei Zhu:** Formal analysis. **Changbing Pu:** Formal analysis. **Dongqi Zhao:** Formal analysis. **Qiong Zhou:** Resources. **Jin Wang:** Supervision, Funding acquisition. **Lijin Lin:** Validation, Supervision. **Dong Liang:** Resources, Funding acquisition, Conceptualization. **Xiulan Lv:** Supervision, Project administration, Funding acquisition, Conceptualization.

## Declaration of competing interest

The authors declare that they have no known competing financial interests or personal relationships that could have influenced the work reported in this paper.

## Data Availability

Data will be made available on request.

## Appendix A Supplementary data

Supplementary material 1 Image 1

Supplementary material 2 Image 2
