# Supplementary material for: Soluble sugar, organic acid and phenolic composition and flavor evaluation of plum fruits
Source: Food Chem X. 2024 Aug 31;24:101790. doi: 10.1016/j.fochx.2024.101790 (PMC11408021; doi:10.1016/j.fochx.2024.101790)
Supplement: Supplementary file 1 — Supplementary material 1 [file mmc1.docx]

**Table S1** Description of characteristics of plums.

| Accession | Type | Locality (City, Province) | Maturity  (date) | Fruit size  (g) | Fruit shape | Peel color | Flesh color | TSS (%) | TA (%) | TSS/TA | Sensory Flavor | | | |
| --- | --- | --- | --- | --- | --- | --- | --- | --- | --- | --- | --- | --- | --- | --- |
|  |  |  |  |  |  |  |  |  |  |  | ^1^score | ^1^sour sweet | ^2^score | ^2^astringency |
| FT | Prunus salicina | Anshun,Guizhou | Mid June | 31.4±4.2 | Near round | yellow green | yellow green | 12.79±0.10 | 0.97±0.02 | 13.18 | 5.50 | sweet-sour | 0.00 | no |
| AGL | Prunus domestica | Ya'an,Sichuan | late July | 78.7±8.1 | Oblate | purple-black | light yellow | 10.01±0.16 | 1.27±0.04 | 7.88 | 2.75 | sour-sweet | 0.00 | no |
| ADLD | Prunus domestica | Wanzhou,Chongqing | late July | 54.9±10.0 | Oblate | prune | light yellow | 11.08±0.16 | 1.01±0.05 | 10.97 | 3.38 | sour-sweet | 0.75 | light |
| CT | Prunus salicina | Ya'an,Sichuan | late July | 45.5±6.3 | Heart | green | yellow green | 12.26±0.20 | 0.98±0.03 | 12.51 | 3.50 | sour-sweet | 0.00 | no |
| FWMG | P.simonii | Ya'an,Sichuan | late July | 106.5±6.8 | Near round | pink | yellow | 12.81±0.16 | 1.58±0.04 | 8.11 | 2.13 | sour | 1.00 | light |
| FT2 | Prunus salicina | Anshun,Guizhou | Mid June | 36.4±4.5 | Near round | yellow green | yellow green | 11.63±0.18 | 0.82±0.02 | 14.19 | 4.25 | sweet-sour | 0.00 | no |
| FLS | Prunus domestica | Ya'an,Sichuan | late July | 99.6±5.2 | Near round | red | yellow | 11.76±0.20 | 1.56±0.12 | 7.54 | 2.25 | sour | 0.13 | light |
| GZ | Prunus domestica | Wanzhou,Chongqing | late August | 94.2±6.8 | Near round | pink | yellow | 11.91±0.29 | 0.53±0.01 | 22.48 | 4.13 | sweet sour | 1.13 | medium |
| HYBD | Prunus salicina | Ya'an,Sichuan | late July | 35.2±5.2 | Near round | yellow green | yellow green | 11.76±0.16 | 0.56±0.02 | 20.99 | 4.00 | sour-sweet | 0.13 | light |
| HYHC | Prunus salicina | Ya'an,Sichuan | late July | 34.5±4.0 | Near round | red | yellow | 12.73±0.20 | 0.78±0.02 | 16.32 | 4.63 | sweet-sour | 0.00 | no |
| HHP | Prunus domestica | Ya'an,Sichuan | late July | 102.0±8.0 | Near round | purple black | yellow | 12.59±0.13 | 1.46±0.12 | 8.62 | 2.25 | sour | 0.00 | no |
| HJG | Prunus domestica | Ya'an,Sichuan | late July | 63.6±5.8 | Oblate | purple black | yellow | 8.76±0.12 | 1.56±0.02 | 5.61 | 2.13 | sour | 0.00 | no |
| JDN | Prunus salicina | Ya'an,Sichuan | late July | 81.6±5.6 | Reniform | yellow green | yellow | 10.33±0.12 | 1.17±0.01 | 8.83 | 3.88 | sour-sweet | 0.00 | no |
| MN | Prunus salicina | Ya'an,Sichuan | late August | 71.5±7.3 | Near round | yellow | yellow | 15.07±0.16 | 0.61±0.02 | 24.70 | 5.75 | sweet | 0.00 | no |
| MJ | Prunus salicina | Ya'an,Sichuan | late June | 47.6±5.0 | Near round | pink | yellow | 12.38±0.12 | 0.52±0.01 | 23.81 | 4.50 | sweet-sour | 0.00 | no |
| PT | Prunus salicina | Ya'an,Sichuan | late July | 44.0±3.6 | Reniform | yellow green | yellow | 8.72±0.16 | 0.91±0.04 | 9.58 | 3.75 | sour-sweet | 0.00 | no |
| QC | Prunus salicina | Aba,Sichuan | late July | 48.9±8.0 | Near round | green | yellow green | 11.99±0.16 | 0.87±0.03 | 13.78 | 6.00 | sweet | 0.00 | no |
| JM | Prunus salicina | Chengdu,Sichuan | Mid June | 28.6±3.4 | Near round | yellow | yellow | 14.00±0.17 | 0.42±0.02 | 33.33 | 4.25 | sweet-sour | 0.00 | no |
| QD | Prunus salicina | Aba,Sichuan | Early August | 75.2±6.1 | Near round | green | yellow green | 12.94±0.29 | 0.82±0.04 | 15.78 | 6.00 | sweet | 0.00 | no |
| SL | Prunus domestica | Wanzhou,Chongqing | late August | 121.0±11.2 | Near round | dark red | yellow | 10.20±0.05 | 1.10±0.03 | 9.27 | 2.13 | sour | 0.88 | light |
| SYC | Prunus salicina | Wanzhou,Chongqing | Late May | 10.9±2.0 | Near round | red | yellow | 16.24±0.20 | 0.72±0.02 | 22.55 | 7.00 | sweet | 0.00 | no |
| TH | Prunus salicina | Ya'an,Sichuan | Early July | 37.5±4.6 | Near round | yellow | yellow | 11.17±0.12 | 1.02±0.02 | 10.95 | 4.88 | sweet-sour | 0.00 | no |
| WDK | Prunus salicina | Ya'an,Sichuan | late August | 38.5±5.2 | Near round | green | green | 10.13±0.08 | 0.90±0.02 | 11.26 | 4.13 | sweet-sour | 0.13 | light |
| WT | Prunus  salicina | Ya'an,Sichuan | late July | 35.6±4.1 | Near round | green | green | 11.20±0.21 | 0.45±0.02 | 24.89 | 4.13 | sweet-sour | 0.13 | light |
| WD | Prunus.  simonii | Ya'an,Sichuan | Early July | 88.7±6.8 | Near round | red | red | 20.27±0.20 | 1.30±0.11 | 15.59 | 5.88 | sweet | 1.00 | light |
| WSC | Prunus  salicina | Wushan,Chongqing | Early July | 40.1±4.6 | Near round | green | yellow green | 12.14±0.12 | 0.72±0.03 | 16.87 | 4.75 | sweet-sour | 0.00 | no |
| WYC | Prunus salicina | Ya'an,Sichuan | Early July | 35.1±4.0 | Near round | prune | yellow | 16.72±0.20 | 0.82±0.02 | 20.39 | 6.63 | sweet | 0.00 | no |
| XCH | Prunus salicina | Ya'an,Sichuan | late July | 30.0±4.1 | Near round | red | yellow | 12.86±0.08 | 0.77±0.03 | 16.70 | 5.50 | sweet-sour | 0.00 | no |
| YHL | Prunus salicina | Yibin,Sichuan | late July | 50.0±5.6 | Near round | red | yellow | 12.56±0.04 | 0.66±0.03 | 19.02 | 4.75 | sweet-sour | 0.00 | no |
| YQC | Prunus salicina | kunming,Yunnan | Early July | 45.0±3.2 | Near round | green | green | 11.77±0.10 | 0.65±0.03 | 18.10 | 4.88 | sweet-sour | 0.00 | no |
| ZH | Prunus salicina | Ya'an,Sichuan | Early July | 69.24±6.8 | Heart | pink | yellow | 11.21±0.13 | 1.36±0.10 | 8.25 | 4.50 | sweet-sour | 0.25 | light |
| ZHP | Prunus salicina | Ya'an,Sichuan | Early July | 46.5±5.2 | Near round | prune | yellow | 14.04±0.09 | 0.88±0.06 | 15.96 | 3.25 | sour sweet | 0.00 | no |
| ZTL | Prunus domestica | Ya'an,Sichuan | Early July | 72.2±6.5 | Oblate | red | yellow | 10.38±0.08 | 1.45±0.02 | 7.16 | 2.50 | sour | 1.25 | medium |
| QF | Prunus salicina | Chengdu,Sichuan | Early July | 51.2±5.0 | Near round | red | yellow green | 14.38±0.08 | 0.41±0.02 | 35.07 | 6.00 | sweet | 0.00 | no |
| HF | Prunus salicina | Chengdu,Sichuan | Early July | 49.5±6.3 | Near round | red | yellow green | 14.37±0.13 | 0.52±0.03 | 27.63 | 6.25 | sweet | 0.00 | no |
| CM | Prunus salicina | Chengdu,Sichuan | Early July | 35.5±5.6 | Near round | green | yellow | 11.64±0.29 | 0.56±0.01 | 20.78 | 5.00 | sweet-sour | 0.00 | no |
| HBS | Prunus domestica | Aba,Sichuan | Early July | 72.3±5.4 | Oblate | red | yellow | 9.49±0.05 | 1.06±0.02 | 8.95 | 3.00 | sour-sweet | 0.00 | no |
| HXM | Prunus domestica | Ya'an,Sichuan | late July | 21.8±4.2 | Ellipse | red | pink | 9.64±0.12 | 0.60±0.01 | 16.05 | 2.00 | sour | 0.00 | no |
| JQ | Prunus salicina | Jiuqian,Guizhou | Early July | 13.5±2.0 | Near round | red | yellow | 14.98±0.16 | 0.66±0.02 | 22.70 | 4.38 | sweet-sour | 0.00 | no |
| KX | Prunus salicina | Anshun,Guizhou | Early July | 25.3±4.2 | Near round | green | yellow green | 11.14±0.12 | 0.41±0.02 | 27.17 | 3.38 | sour-sweet | 0.00 | no |
| LV | Prunus salicina | Ya'an,Sichuan | late August | 56.4±6.0 | Near round | red | yellow | 19.12±0.21 | 0.77±0.01 | 24.99 | 6.13 | sweet | 0.00 | no |
| QCA | Prunus salicina | Ya'an,Sichuan | late June | 32.0±4.0 | Near round | green | green | 7.77±0.20 | 0.44±0.01 | 17.65 | 3.00 | sour-sweet | 0.00 | no |
| SH | Prunus salicina | kunming,Yunnan | late June | 37.4±3.5 | Heart | red | pink | 12.26±0.11 | 0.75±0.01 | 16.34 | 3.75 | sour-sweet | 0.00 | no |
| SYC22 | Prunus salicina | Ya'an,Sichuan | Late May | 20.0±4.0 | Near round | red | yellow | 14.19±0.21 | 0.80±0.01 | 17.74 | 5.88 | sweet | 0.00 | no |
| YUH | Prunus salicina | Ya'an,Sichuan | Early July | 69.3±7.5 | Near round | yellow | yellow | 11.56±0.41 | 0.68±0.02 | 16.89 | 4.75 | sweet-sour | 0.00 | no |
| ZTL2 | Prunus domestica | Ya'an,Sichuan | late July | 62.0±8.4 | Oblate | prune | yellow | 10.31±0.21 | 1.06±0.02 | 9.73 | 3.38 | sour-sweet | 1.38 | medium |
| MHC | Prunus salicina | Aba,Sichuan | late August | 35.2±5.3 | Near round | prune | yellow | 12.77±0.20 | 0.85±0.02 | 15.02 | 5.38 | sweet-sour | 0.00 | no |
| MHD | Prunus salicina | Aba,Sichuan | late August | 42.5±5.0 | Near round | prune | yellow | 13.39±0.08 | 0.86±0.05 | 15.57 | 5.50 | sweet-sour | 0.00 | no |
| MQC | Prunus salicina | Aba,Sichuan | late August | 46.5±4.5 | Near round | green | yellow | 12.89±0.08 | 0.88±0.02 | 14.65 | 5.38 | sweet-sour | 0.00 | no |
| MQD | Prunus salicina | Aba,Sichuan | late August | 70.5±8.2 | Near round | green | yellow | 13.56±0.33 | 1.10±0.04 | 12.32 | 5.50 | sweet-sour | 0.00 | no |
| MN8 | Prunus salicina | Ya'an,Sichuan | late August | 72.5±6.1 | Near round | yellow | yellow | 16.17±0.21 | 0.88±0.04 | 18.37 | 6.13 | sweet | 0.00 | no |
| BT | Prunus salicina | Anshun,Guizhou | late June | 31.4±3.5 | Near round | yellow green | yellow green | 13.23±0.11 | 0.95±0.02 | 13.93 | 4.63 | sweet-sour | 0.00 | no |
| HBK | Prunus salicina | Ya'an,Sichuan | Early July | 35.9±6.2 | Near round | green | yellow green | 16.80±0.09 | 0.69±0.03 | 24.35 | 4.63 | sweet-sour | 0.00 | no |
| QYN | Prunus salicina | Ya'an,Sichuan | late July | 65.2±6.0 | Reniform | green | yellow green | 14.24±0.20 | 0.43±0.01 | 33.11 | 4.38 | sweet-sour | 0.00 | no |
| MJY | Prunus salicina | Aba,Sichuan | Early Sep | 42.3±5.1 | Near round | red | yellow | 13.24±0.20 | 0.87±0.04 | 15.22 | 5.63 | sweet | 0.00 | no |
| HF1 | Prunus salicina | Aba,Sichuan | late July | 36.5±4.3 | Near round | red | yellow | 14.19±0.21 | 1.10±0.05 | 12.90 | 5.75 | sweet | 0.00 | no |
| MEI21 | Prunus domestica | Ya'an,Sichuan | late July | 28.5±3.5 | Ellipse | prune | yellow | 10.13±0.12 | 0.82±0.03 | 12.36 | 3.75 | sour-sweet | 0.00 | no |
| JX | Prunus salicina | kunming,Yunnan | late July | 30.5±6.2 | Heart | red | red | 11.00±0.09 | 1.10±0.06 | 10.00 | 3.88 | sour-sweet | 0.00 | no |
| FR | Prunus salicina | kunming,Yunnan | Early June | 83.9±6.5 | Heart | red | red | 11.24±0.20 | 0.85±0.02 | 13.23 | 3.88 | sour-sweet | 0.00 | no |
| DHP | Prunus salicina | Yibin,Sichuan | late July | 135.0±15.2 | Near round | red | light yellow | 11.53±0.33 | 0.65±0.02 | 17.74 | 4.25 | sweet-sour | 1.63 | medium |
| DBL | Prunus salicina | Yibin,Sichuan | Early July | 35.2±6.9 | Near round | green | yellow green | 10.68±0.54 | 0.34±0.02 | 31.41 | 4.38 | sweet-sour | 0.00 | no |
| MGH | Prunus salicina | kunming,Yunnan | Early July | 44.1±5.8 | Near round | red | red | 10.44±0.12 | 1.02±0.12 | 10.24 | 4.50 | sweet-sour | 0.00 | no |
| MEIN | Prunus domestica | Aba,Sichuan | late July | 21.8±4.6 | Ellipse | prune | light yellow | 11.59±0.29 | 0.69±0.16 | 16.80 | 3.50 | sour-sweet | 0.00 | no |
| MEIF | Prunus domestica | Aba,Sichuan | late July | 29.6±5.4 | Ellipse | prune | light yellow | 12.27±0.20 | 0.70±0.03 | 17.52 | 3.00 | sour-sweet | 0.00 | no |
| HZF | Prunus salicina | Wanzhou,Chongqing | late August | 78.2±7.4 | Near round | prune | light yellow | 12.57±0.22 | 0.62±0.01 | 20.27 | 5.38 | sweet-sour | 1.25 | medium |
| WHB | Prunus domestica | Wanzhou,Chongqing | late August | 85.6±10.2 | Oblate | purple black | yellow | 13.66±0.21 | 1.10±0.02 | 12.41 | 4.63 | sweet-sour | 1.88 | medium |
| KLD | P.simonii | Yibin,Sichuan | Early July | 80.2±6.8 | Near round | red | pink | 12.17±0.12 | 1.10±0.03 | 11.06 | 3.63 | sour-sweet | 0.00 | no |
| ZXT | Prunus salicina | Chengdu,Sichuan | Early July | 65.8±7.3 | Oblate | orange | yellow | 10.00±0.09 | 0.51±0.02 | 19.57 | 4.38 | sweet-sour | 0.00 | no |
| ZUO | Prunus salicina | Chengdu,Sichuan | Early July | 42.1±6.3 | Near round | red | yellow | 15.97±0.16 | 0.68±0.02 | 23.48 | 5.50 | sweet-sour | 1.00 | light |
| HBL | Prunus salicina | Chengdu,Sichuan | late July | 35.6±4.5 | Oblate | purple black | yellow | 9.83±0.21 | 0.61±0.04 | 16.12 | 3.13 | sour-sweet | 1.00 | light |
| MGH2 | Prunus salicina | Chengdu,Sichuan | late June | 28.3±4.2 | Near round | red | red | 10.07±0.16 | 1.00±0.06 | 10.07 | 3.63 | sour-sweet | 0.00 | no |
| TXL | Prunus salicina | kunming,Yunnan | late June | 30.1±3.5 | Heart | red | pink | 7.93±0.03 | 0.51±0.10 | 15.56 | 4.13 | sweet-sour | 0.25 | light |
| ZYL | P.cerasifera atropurpurea | Yibin,Sichuan | late June | 18.5±5.2 | Near round | red | red | 8.10±0.08 | 0.66±0.05 | 12.25 | 2.63 | sour-sweet | 0.25 | light |
| YOU | Prunus salicina | Chengdu,Sichuan | late June | 50.2±6.5 | Oblate | prune | yellow | 13.90±0.12 | 0.66±0.10 | 21.06 | 4.88 | sweet-sour | 0.88 | light |
| KLD2 | Prunus domestica | Yibin,Sichuan | Early July | 65.2±4.8 | Near round | red | red | 7.93±0.08 | 0.95±0.10 | 8.35 | 2.13 | sour | 0.38 | light |
| HJN | Prunus salicina | Yibin,Sichuan | late July | 80.5±7.5 | Reniform | yellow | yellow | 15.37±0.41 | 0.45±0.06 | 34.15 | 4.88 | sweet-sour | 0.00 | no |
| ALS | Prunus domestica | Chengdu,Sichuan | late July | 67.8±6.4 | Near round | dark blue | dark red | 8.80±0.21 | 0.32±0.02 | 27.50 | 4.88 | sweet-sour | 0.75 | light |
| SXP | Prunus domestica | Chengdu,Sichuan | late July | 58.5±6.8 | Near round | red | yellow | 9.43±0.43 | 0.53±0.05 | 17.93 | 4.50 | sweet-sour | 0.13 | light |
| ZH | Prunus salicina | Chengdu,Sichuan | late July | 60.2±4.3 | Oblate | red | yellow | 10.57±0.33 | 0.45±0.02 | 23.48 | 4.63 | sweet-sour | 0.38 | light |
| YG | Prunus salicina | Chengdu,Sichuan | late July | 50.5±6.3 | Reniform | yellow | yellow | 9.13±0.61 | 0.49±0.02 | 18.83 | 4.63 | sweet-sour | 0.00 | no |
| MS | Prunus salicina | Chengdu,Sichuan | late July | 20.6±4.1 | Near round | green | yellow green | 9.30±0.33 | 0.66±0.02 | 14.20 | 4.75 | sweet-sour | 0.00 | no |
| GR | Prunus salicina | Chengdu,Sichuan | Early July | 45.2±5.6 | Heart | orange | yellow | 10.33±0.12 | 0.40±0.02 | 25.83 | 4.25 | sweet-sour | 0.00 | no |
| SJ | Prunus salicina | Aba,Sichuan | Early July | 28.6±5.2 | Near round | red | pink | 15.80±0.08 | 0.57±0.01 | 27.92 | 6.13 | sweet | 0.00 | no |
| HHL | Prunus salicina | Chengdu,Sichuan | late August | 88.5±6.4 | Reniform | yellow green | yellow green | 14.67±0.21 | 0.45±0.01 | 32.59 | 5.88 | sweet | 0.00 | no |
| HBS2 | Prunus domestica | Chengdu,Sichuan | late July | 79.2±5.8 | Oblate | dark red | yellow | 10.77±0.20 | 1.22±0.01 | 8.83 | 4.50 | sweet-sour | 0.63 | light |
| QMG | Prunus salicina | Yibin,Sichuan | Early Sep | 81.2±8.5 | Oblate | dark blue | yellow | 9.93±0.03 | 1.65±0.11 | 6.02 | 2.00 | sour | 1.75 | medium |

Note: TSS, Total soluble solid content. TA, titratable acid. TSS/TA, the ratio of TSS to TA. Fruit-related traits were measured based on the phenotyping protocol of plums. Sensory flavors include sweet-sour and astringency.

**Table S5** Cardinality test for sensory evaluation and grade rating.

|  | Flavor rating | | | |  | χ^2^ | *P* |
| --- | --- | --- | --- | --- | --- | --- | --- |
|  | Sweet | Sweet-sour | Sour | Sour-sweet | Total |  |  |
| Sensory evaluation | 9 | 21 | 37 | 19 | 86 | 8.891 | **0.03*** |
| Grade rating | 19 | 29 | 28 | 10 | 86 |  |  |
| Total | 28 | 50 | 65 | 29 | 172 |  |  |

Note: **p* < 0.05, ***p* < 0.01.

**Table S6** Correlation of phenolic components with astringency values

| Component | PAsB1 | PAsB2 | PAsC1 | Epicatechin | Catechin | Rutin | Quercetin | Gallic | Chlorogenic | Total Phenolics(peel) | Total Phenolics(flesh) |
| --- | --- | --- | --- | --- | --- | --- | --- | --- | --- | --- | --- |
| Astringency | 0.09 | **0.356**** | **0.422**** | **0.641**** | -0.05 | 0.248* | 0.08 | **0.476**** | 0.17 | **0.706**** | 0.424** |

Note: **p* < 0.05, ***p* < 0.01.

**Table S7** Grading of flavor character evaluation of 86 plum accessions

| Types | Number | Proportion | Fructose | Glucose | Sucrose | TSS | TSS/TA | TA | Malic acid | Total phenolics | Evaluation |
| --- | --- | --- | --- | --- | --- | --- | --- | --- | --- | --- | --- |
| Ⅰ | 36 | 41.86 | 12.57 | 16.24 | 23.93 | 11.64 | 20.00 | 0.61 | 727.91 | 118.25 | sucrose-dominant |
| Ⅱ | 13 | 15.12 | 21.32 | 30.69 | 21.58 | 15.63 | 25.66 | 0.66 | 791.34 | 115.87 | comprehensive high-sugar |
| Ⅲ | 23 | 26.74 | 16.27 | 25.00 | 20.77 | 11.67 | 12.59 | 0.96 | 630.60 | 240.86 | comprehensive high-phenolic |
| Ⅳ | 14 | 16.28 | 12.24 | 20.66 | 10.30 | 10.86 | 8.99 | 1.27 | 1110.48 | 173.69 | hyperacidic |


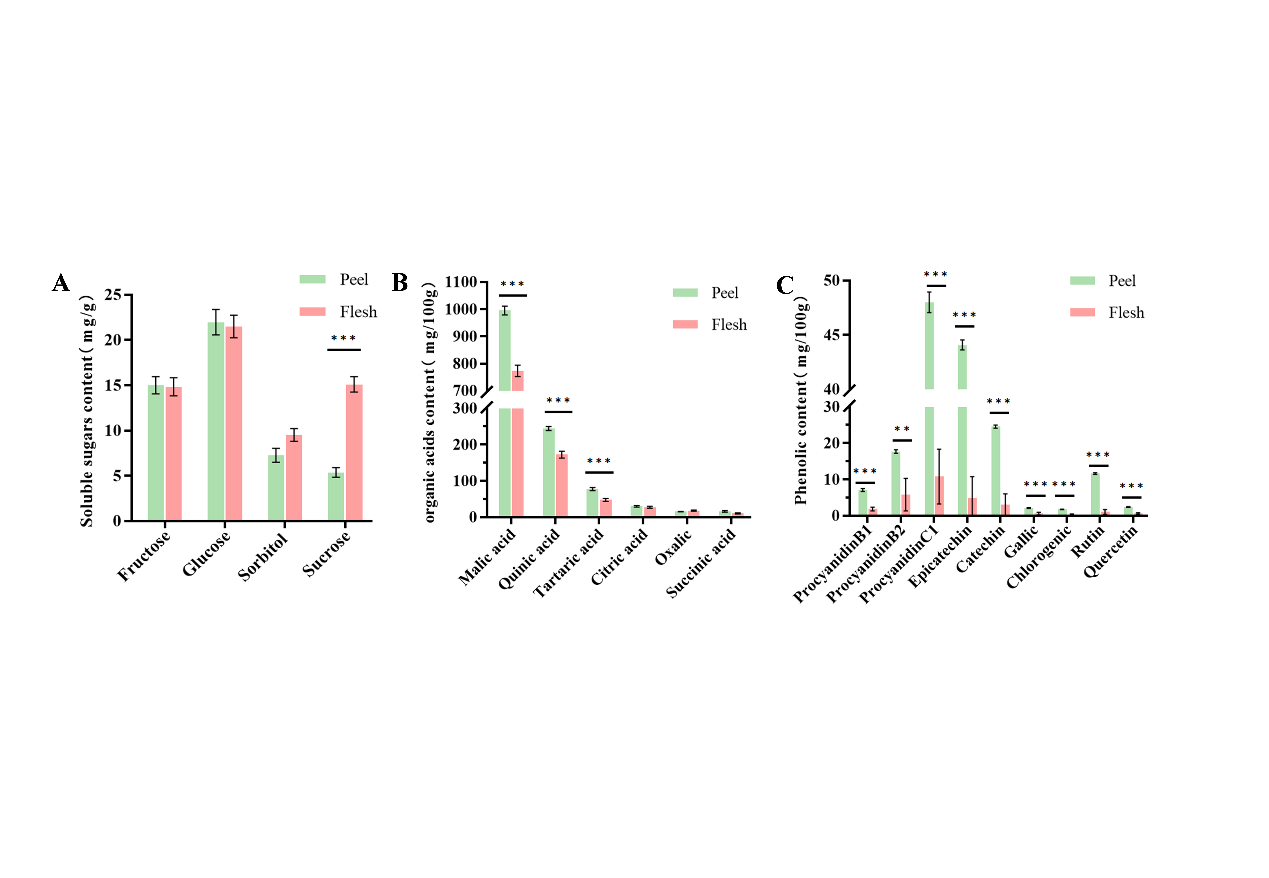


**Fig.S1** Comparison of distribution of sugar (A), organic acid (B) and phenolic contents (C) between peel and flesh in plum fruits. *, **, and *** indicate significance at 0.05, 0.01, and 0.001 level, respectively.


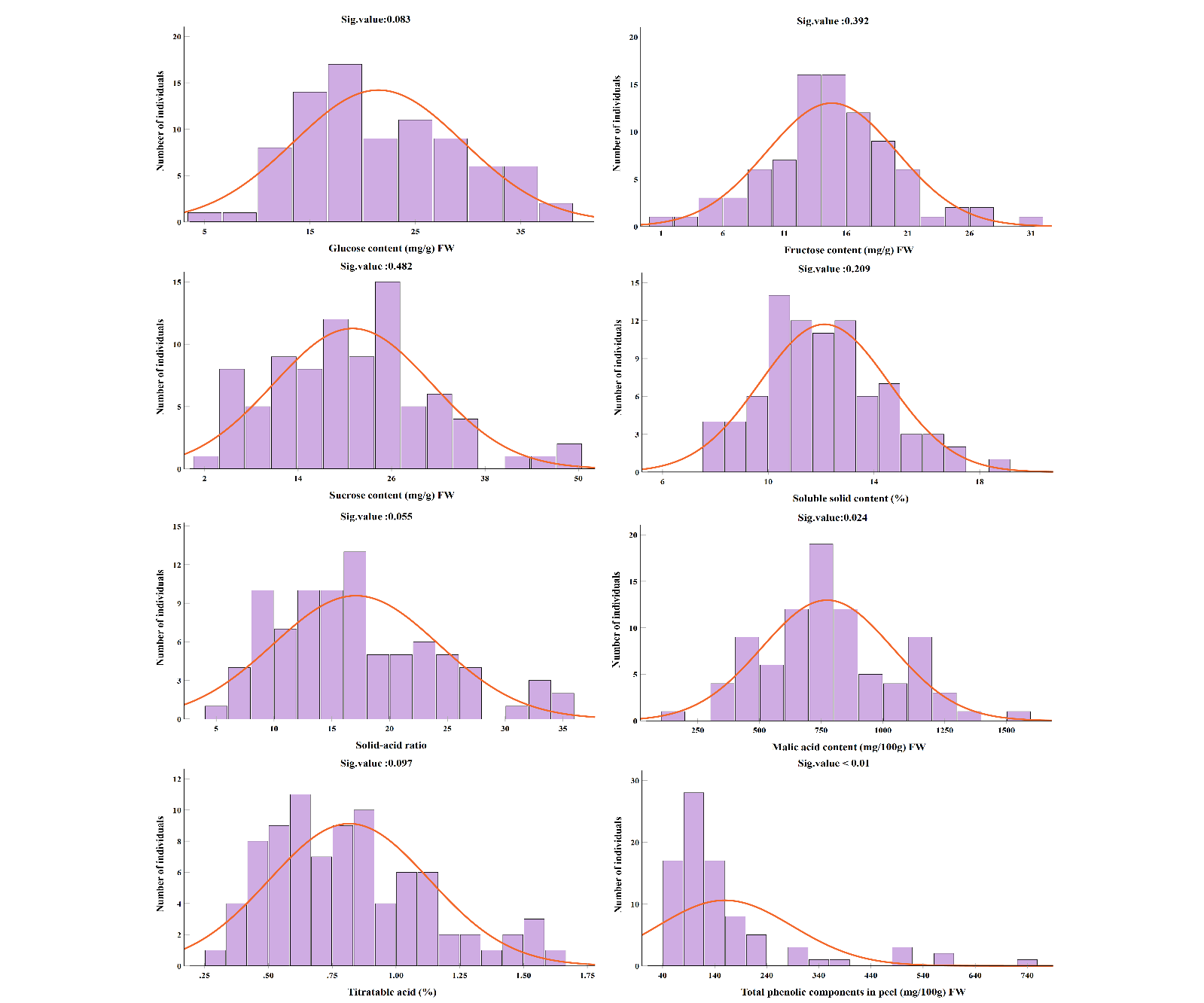


**Fig.S2** Normal distribution of eight fruit flavor indices in plum fruits.

Note: Sig. value, significance. Sig. value ≥ 0.05 indicates a normal distribution.

**Table S8** Mathematical terms

| Mathematical terms | notes |
| --- | --- |
| χ^2^ | Chi-Squared Test |
| *P* | *P*- value |
| Sig. value | significance |
| FW | Fresh weight |
| ◦Brix % | Degrees Brix |
| Pro % | Proportion |
| Cv | coefficients of variation |
| Mean | average value |
| SD | (statistics) standard deviation |
| R | Correlation coefficient |
